# An unusual presentation of subacute *Haemophilus parainfluenzae* endocarditis in a low-risk woman treated by minimally invasive mitral valve repair: a case report

**DOI:** 10.1186/s43044-024-00482-6

**Published:** 2024-05-06

**Authors:** Younus Qamar, Ahmed Shazly, Amna Qamar, Heraa Islam, Hannah Yonis, Haytham Sabry

**Affiliations:** 1 Department of Cardiothoracic Surgery, The Essex Cardiothoracic Centre, Basildon and Thurrock University Hospital, Mid and South Essex NHS Foundation Trust, Nethermayne, Basildon, SS16 5NL UK; 2 Department of Cardiothoracic Surgery, John Radcliffe Hospital, Oxford University Hospitals NHS Foundation Trust, Oxford, UK; 3 Department of General Surgery, Queen’s Hospital, Barking, Havering and Redbridge University Hospitals NHS Trust, Romford, UK; 4 Department of Cardiothoracic Surgery, Liverpool Heart and Chest Hospital Foundation Trust, Liverpool, UK

**Keywords:** *Haemophilus parainfluenzae* endocarditis, Mitral valve perforation, Janeway lesion, Splinter haemorrhage, Mitral valve repair, Right anterior thoracotomy, Case report

## Abstract

**Background:**

HACEK endocarditis is usually insidious and can often be difficult to diagnose due to the slow-growing nature of the organisms. This report presents our experience in treating a patient with *Haemophilus parainfluenzae* endocarditis.

**Case presentation:**

We describe the case of a previously fit and well 23 year-old woman who presented to her local emergency department with a four-week history of persistent febrile illness. She had associated nausea, vomiting, and lethargy. This was preceded by an episode of mucopurulent rhinorrhoea. She was treated empirically with oral amoxicillin for a putative diagnosis of rhinosinusitis. Initially, her symptoms abated, however, she was readmitted with high fevers and a new pansystolic murmur. Transthoracic echocardiography revealed a large, mobile, echogenic mass, tethered to the posterior mitral valve leaflet (PMVL) and mild mitral regurgitation (MR). On examination, she had multiple non-tender, erythematous macules on the plantar surface of her feet, consistent with Janeway lesions. Two separate blood cultures grew *H. parainfluenzae*. Infectious diseases recommended a four-week course of intravenous ceftriaxone. Transesophageal echocardiography demonstrated a perforation within the P3 segment of the PMVL. Subsequently, the patient underwent mitral valve repair surgery with an uneventful recovery.

**Conclusions:**

Our case highlights the importance of promptly diagnosing HACEK endocarditis. A prolonged course of antibiotic therapy can be lifesaving, and surgery is often necessary to address complications such as perforation within the mitral valve leaflets. In our patient, we were able to perform a sliding P2 leaflet plasty for good quality repair of the mitral valve, through a minimally invasive right anterior thoracotomy.

## Background

The annual incidence of bacterial infective endocarditis (BIE) is 3–10 cases per 100,000 people [1]. The term endocarditis refers to inflammation of the inner layer of the heart wall, or the endocardium. In BIE, the endocardium lining heart valves are typically affected. The classic organisms implicated in > 90% of culture-positive BIE are *Streptococci*, *Staphylococci*, and *Enterococci* [2].

The HACEK group of bacteria (*Haemophilus spp., Aggregatibacter spp., Cardiobacterium hominis*, *Eikenella corrodens*, and *Kingella spp.*) cause approximately 3% of all cases of BIE [3]. They are gram-negative organisms that colonise the oropharyngeal, upper respiratory, and urogenital mucosae in humans [3, 4]. They usually cause BIE in individuals with predisposing conditions, including bicuspid aortic valve, mitral valve prolapse, congenital cardiac malformations, rheumatic heart disease, prosthetic valves, and prior native valve endocarditis. Intravenous drug use, poor dental hygiene and long-term venous access are recognised risk factors. Clinical presentation of HACEK endocarditis is often subacute or insidious owing to the slow-growing nature of the bacteria. Third-generation cephalosporin (e.g., ceftriaxone) is the antibiotic of choice; 80–90% of patients respond to cephalosporin therapy, with or without surgical intervention [3, 5, 6].

*Haemophilus parainfluenzae* belongs to the family of HACEK bacteria and represents 27–35% of all HACEK endocarditis [5, 7–9]. It has a predilection for young women (mean age 36 years) with an insidious presentation; the average interval between symptom onset and diagnosis is 34–37 days. The delay in diagnosis explains the typical echocardiography findings of a large (> 10 mm) vegetation, which has a high tendency for systemic embolization, causing stroke, splenic or renal infarction, Janeway lesions, and nail-bed (splinter) haemorrhages [10]. The vegetations are destructive and often cause perforation of the valve leaflets; the mitral valve is most commonly affected [11].

Herein, we present a case of a 23-year-old woman, who presented with a four-week history of persistent febrile illness. Positive blood cultures and presence of a large, mobile vegetation identified by echocardiography, confirmed the diagnosis of *H. parainfluenzae* endocarditis.

## Case presentation

A previously fit and well 23-year-old woman presented to her local emergency department with a 4-week history of persistent febrile illness. She had associated nausea, vomiting, and lethargy. She had no history of recent contact with unwell individuals or travel abroad. She denied any recent dental procedures or history of substance misuse. She had no comorbidities and was immunocompetent. She did not take any regular medications, except for an over-the-counter contraceptive pill. A week prior to her admission, she had an episode of mucopurulent rhinorrhoea, which was treated empirically with oral amoxicillin for suspected rhinosinusitis. Her symptoms were partially ameliorated by the antibiotics; however, she was readmitted with unremitting nausea, vomiting, and pyrexia.

On admission, her blood pressure was 92/59 mmHg, pulse rate was 103/min, and respiratory rate was eighteen breaths/min. She was febrile with a temperature of 39.4 °C, and her oxygen saturation was 95% on room air. She was alert and oriented to time, place, and person, and was able to provide a history. On auscultation of the precordium, a grade 3/6 pansystolic murmur was audible at the cardiac apex, radiating into the left axilla. Electrocardiogram showed sinus tachycardia (105 beats/min) with a PR interval of 154 ms and a narrow QRS complex.

Multiple antigenic and molecular SARS-CoV-2 tests were negative. Additionally, a HIV antigen/antibody screen and an anti-neutrophil cytoplasmic antibody (ANCA) test were both negative. Laboratory tests were significant for neutrophilic leukocytosis with a white-cell count (WCC) of 26.1 × 10^9^/L and a neutrophil count of 23.6 × 10^9^/L. The patient was also anaemic with a serum haemoglobin level of 102 g/L and a mean cell volume (MCV) of 86 femtolitres (fL), consistent with normocytic anaemia. She did not report any history of menorrhagia or obvious gastrointestinal (GI) blood loss. Erythrocyte sedimentation rate (ESR) and C-reactive protein (CRP) were markedly elevated at 105 and 187.4, respectively.

Plain chest X-ray showed clear lung fields with no focus of consolidation. Urinalysis was negative for nitrates and leukocytes. Two sets of blood cultures were obtained, and empirical treatment with doxycycline was commenced.

**Fig. 1:**
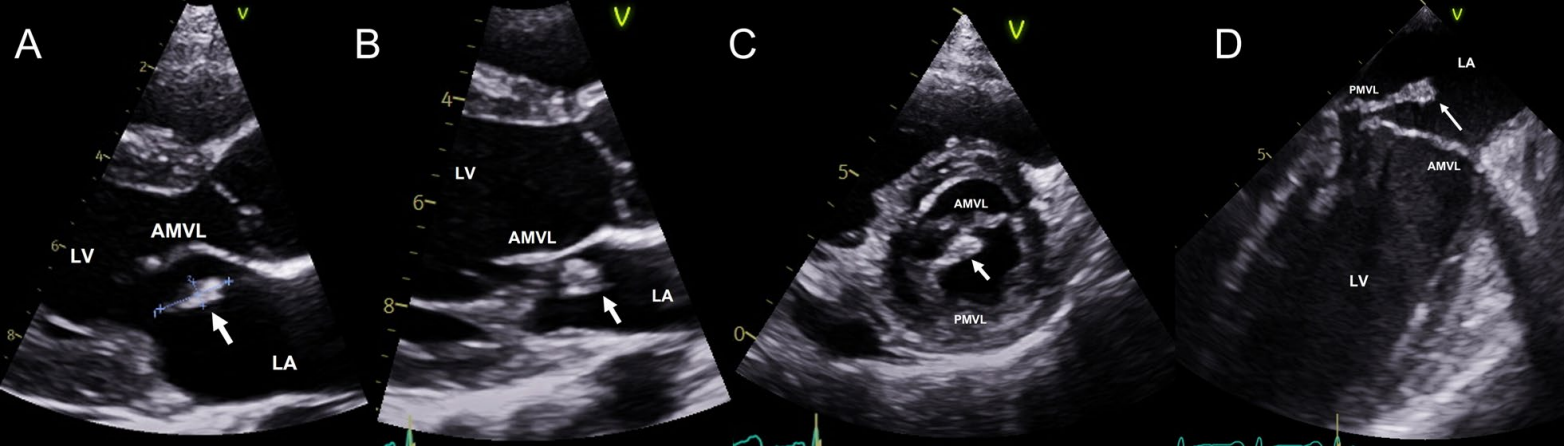
Sequential preoperative echocardiographic images. Legend: Panel **A**: 2D TTE parasternal long axis view showing an echogenic mass, measuring 1.48 × 0.53 cm. Panel **B**: 2D TTE parasternal long axis view showing vegetation on the PMVL. Panel **C**: 2D TTE parasternal short axis view showing vegetation on the medial (P3) segment of the PMVL. Panel **D**: 2D TOE ME two-chamber view showing vegetation attached to the PMVL. 2D, two-dimensional; TTE, transthoracic echocardiography; PMVL, posterior mitral valve leaflet; TOE, transesophageal echocardiography; ME, mid-esophageal

Transthoracic echocardiography (TTE) showed an oscillating mass, measuring 1.48 × 0.53 cm, on the atrial surface of the posterior mitral valve leaflet (PMVL), near the posteromedial commissure (Fig. 1). There was an eccentric jet of mild MR with preserved left ventricular function.

During the first five days of hospitalisation, the patient continued to have daily fevers with a maximum temperature of 38.3 °C. She noticed multiple, erythematous, non-tender macules (2–3 mm) appearing on the sole of her right foot, consistent with Janeway lesions. We noted that she also had splinter or nail-bed haemorrhages. After four days of incubation, blood cultures grew gram-negative rods or bacilli from the aerobic bottles, suspicious for *Haemophilus* species.

Subsequently, transesophageal echocardiography (TOE) confirmed the TTE findings and showed billowing of the PMVL with a perforation within the P3 segment (Fig. 1). The following day, blood cultures speciated *H. parainfluenzae*. We consulted our microbiology colleagues, who advised us to switch from oral doxycycline to 2 g of intravenous ceftriaxone daily.

Eventually, a diagnosis of infective endocarditis (IE) was established based on the Modified Duke criteria, with two major criteria (typical microorganisms consistent with IE from two separate blood cultures and presence of a vegetation on TTE/TOE). Fortunately, our patient responded well to the cephalosporin therapy; her symptoms, including headaches and nausea, resolved, and she remained apyrexial for the remainder of her hospital stay. Serial laboratory testing demonstrated a gradual decline in CRP with normalisation of the WCC. Additionally, all subsequent blood cultures were negative with no growth of any organism.

This case was discussed in our dedicated IE multidisciplinary team meeting, where the consensus was to proceed with urgent surgical intervention with the aim of repairing the mitral valve and preserving native tissues.

Approximately four-weeks following initial presentation, the surgery was performed under general anaesthesia with the use of a double-lumen endotracheal tube. Following systemic heparinization, the right femoral artery and vein were cannulated using a Seldinger technique under TOE guidance. Cardiopulmonary bypass (CPB) was established, and surgical access was obtained by performing a 4-cm right mini-thoracotomy incision in the fourth intercostal space (ICS). An Alexis soft tissue retractor was inserted. A 3D endoscope was introduced via a trocar in the same ICS as the minithoracotomy for visualisation. Additional ports were created to introduce the cardioplegia line, the Chitwood clamp, and a left atrial vent. Following cardioplegic arrest, the left atrium was opened parallel to the interatrial groove and the mitral valve was inspected: there was a perforation within the P3 segment of the PMVL with a large vegetation attached to the subvalvular apparatus. The P3 segment was excised along with the posteromedial commissure. The P2 segment was detached and a sliding plasty was performed using continuous 5/0 Prolene sutures. The posteromedial commissure was plicated using 3/0 Ethibond sutures. The subvalvular apparatus was extensively debrided, and all macroscopic infected tissue was excised. All specimens obtained were sent for microscopy, cultures, and sensitivity. The left atrium was closed using 3/0 Prolene sutures and the heart de-aired. After rewarming, the patient was gradually weaned-off CPB with the heart beating spontaneously in normal sinus rhythm.

**Fig. 2:**
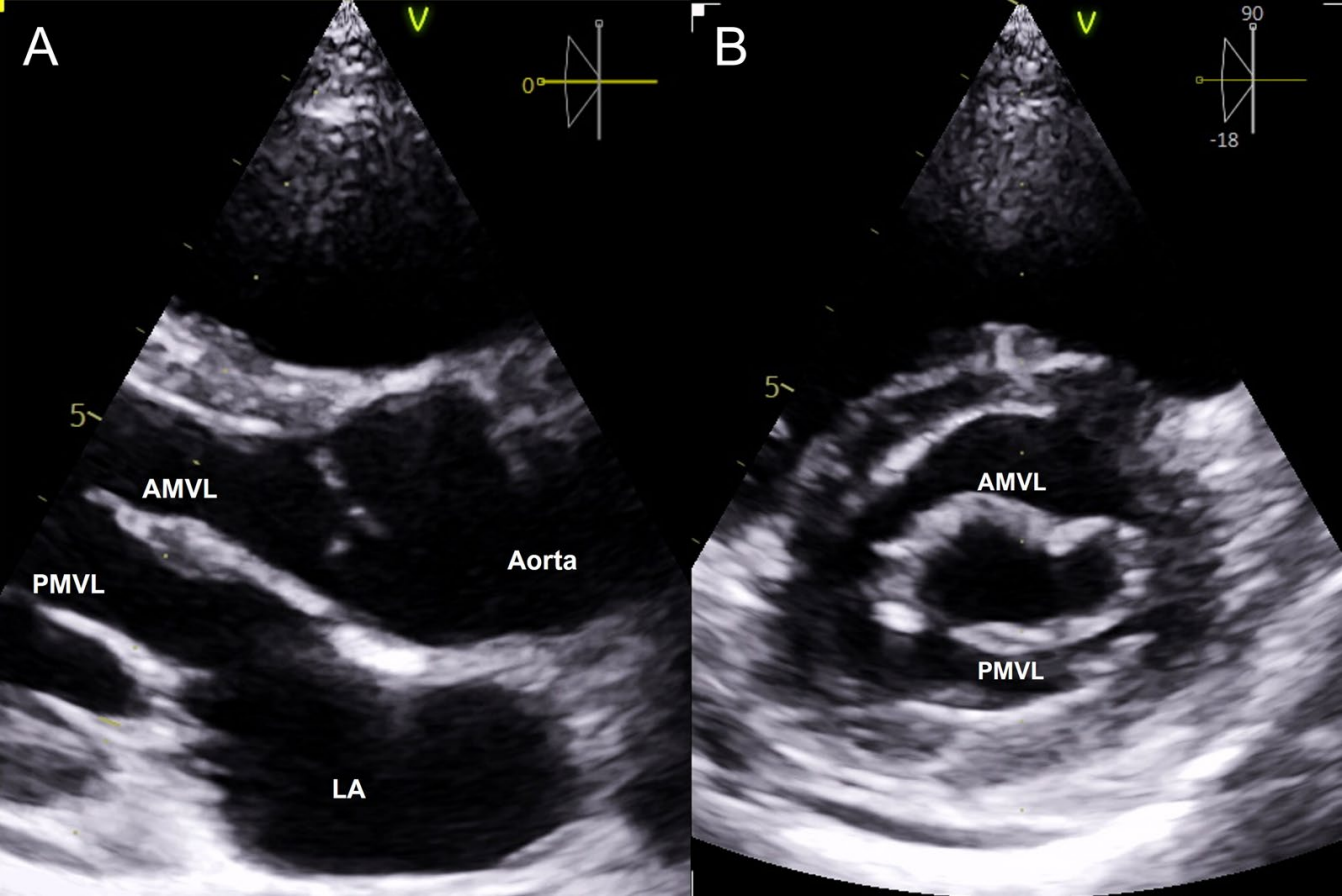
Sequential postoperative echocardiographic images. Legend: Panel **A**: 2D TTE parasternal long axis view showing intact AMVL and PMVL without any vegetations. Panel **B**: 2D TTE parasternal short axis view of the MV leaflets with no vegetations noted on the PMVL. 2D, two-dimensional; TTE, transthoracic echocardiography; AMVL, anterior mitral valve leaflet; PMVL, posterior mitral valve leaflet; MV, mitral valve

The patient was transferred to the intensive care unit (ICU) in a stable condition, and her immediate postoperative course was uneventful. The postoperative TTE showed a well-functioning mitral valve repair with only trivial residual MR (Fig. 2). She completed her four-week course of IV ceftriaxone and continues to do well.

## Discussion

In our patient, we suspect that the source of bacteremia was a recent upper respiratory or sinus infection, given her symptoms of BIE were heralded by mucopurulent rhinorrhoea. *Haemophilus parainfluenzae* bacteremia has previously been reported in association with maxillary sinusitis, and was complicated by mitral valve endocarditis, which presented with multiple cerebral emboli [12]. Although in our patient, there were no clinical signs of a stroke, she had a head computed tomography (CT) scan to rule out a clinically silent cerebral embolus. Typically, the vegetations in *Haemophilus spp.* endocarditis are large, and consequently, have a high propensity for systemic embolization, including to the brain, kidneys, spleen and liver; systemic embolization occurs in approximately 70% of patients who are subsequently diagnosed with *H. parainfluenzae* endocarditis [5, 11]. In our patient, we noticed non-tender, macular lesions of the sole of her right foot, which were consistent with Janeway lesions and splinter haemorrhages. The likelihood for embolization is directly related to the size and filamentous morphology of the vegetations in *H. parainfluenzae* endocarditis [13]. In one series, vegetations > 10 mm were reportedly three times as likely to embolize than those that were < 10 mm in size [10].

Our patient had other features of *H. parainfluenzae* endocarditis: it usually affects young people, between the ages of 20–30, has a female preponderance, and a predilection for the mitral valve; owing to its high virulence and slow-growing nature, results in destruction of the mitral valve apparatus [11, 14]. Fortunately, in our case, the perforation was limited to the P3 segment of the PMVL, allowing for cardiac surgeons to perform a Carpentier sliding leaflet plasty for mitral valve repair. Notably, the mitral valve is the most commonly affected valve in *H. parainfluenzae* endocarditis, followed by the aortic valve [11].

Our patient also had some atypical findings: firstly, it is unusual for BIE to afflict previously healthy, native heart valves, especially in the absence of risk factors, such as intravenous drug abuse, or a history of rheumatic or congenital heart disease [1, 5, 15]. However, in one series, > 50% of patients diagnosed with *H. parainfluenzae* endocarditis had no underlying valvulopathy [16]. Moreover, our patient was found to be anaemic on serial laboratory tests with a normal mean corpuscular volume (MCV), which is consistent with normocytic anaemia. She had no history of menorrhagia, nor had she noticed any frank blood loss. Our patient was also symptomatic with complaints of unremitting fatigue, palpitations, and pallor; whilst these symptoms may be attributed to the underlying bacteremia, anaemia would certainly have further exacerbated them.

This case highlights the role of transesophageal echocardiography in the investigation and management of mitral valve endocarditis. Due to its proximity to the left atrium and the mitral valve, a TOE is the most sensitive imaging modality to identify vegetations, any damage to the mitral valve apparatus and study the haemodynamic consequences (regurgitation or perforation). In our patient, although a TTE showed vegetation on the PMVL, it failed to demonstrate perforation of the P3 segment, which was identified from the subsequent TOE. clinically, this was an important finding because BIE complicated by a destructive penetrating lesion is an indication for surgical intervention.

In our patient, there were several indications for surgery: left-sided BIE caused by a highly resistant micro-organism, complicated by destructive penetrating lesion with evidence of persistent bacteremia or fever lasting for > 5 days after initiating antibiotic therapy, presenting with recurrent emboli and persistent vegetation despite appropriate antibiotics. Importantly, in our case, urgent surgery was deemed necessary since our patient had native valve endocarditis (NVE) and had mobile vegetation > 10 mm with clinical evidence of embolic phenomena (Janeway lesions and splinter haemorrhages) despite appropriate antibiotics.

Furthermore, TOE has a role in preoperative planning. In this case, the identification of the perforated P3 segment of PMVL in a young female patient, allowed surgeons to counsel the patient on the feasibility for repair and possibility for replacement, either with a bioprosthetic or mechanical valve. If a repair were not feasible or successful, a mechanical prosthesis would warrant lifelong anticoagulation with Warfarin, which is a teratogenic agent. This can have lasting implications with regards to conception for a young woman of reproductive age, since warfarin handling can be challenging perinatally.

This case reiterates the difficulty in diagnosing HACEK endocarditis due to the slow-growing nature of these organisms. Clinically, this translates into a delay in initiating appropriate antibiotics and worse outcomes (e.g., multiple systemic emboli, perforation or destruction of valve apparatus, abscess formation, lethal arrhythmias). The average incubation period for *H. parainfluenzae* is 5 days; in our case, we suspect that a high bacterial inoculum, which was reflected by the severity of our patient’s illness, resulted in quicker speciation [5, 11, 12, 14].

The treatment for *H. parainfluenzae* endocarditis is a 4-week course of intravenous ceftriaxone in NVE and a 6-week course for prosthetic valve endocarditis (PVE). However, antibiotics alone are ineffective in patients with BIE who present with valvular dysfunction resulting in heart failure, complicated by heart block, annular or aortic abscess, destructive vegetations, persistent bacteremia or fever > 5–7 days despite appropriate antibiotic therapy, recurrent emboli or vegetations > 10 mm in length. These factors are invariably linked to a protracted course of BIE with haemodynamic instability and reduced life expectancy. Therefore, timely surgical intervention can prolong survival and improve long-term outcomes [17]. Mitral valve repair (MVr), compared to replacement, is associated with decreased in-hospital and long-term mortality. Additionally, MVr is also associated with decreased recurrence of BIE and overall need for reoperation [18]. However, the result of a MVr is dependent on the experience of the operating surgeon, the extent of valve damage, which is more severe in cases of MVr. Importantly, patients who are considered for replacement tend to be hemodynamically and clinically more unstable in comparison to those who are offered MVr.

The indications for and timing of surgery for patients with BIE have been outlined in the 2023 European Society of Cardiology (ESC), the 2019 American Association for Thoracic Surgery (AATS), and the 2020 American College of Cardiology (ACC) guidelines. Briefly, there are three main indications for surgery in acute BIE: heart failure, uncontrolled infections, and prevention of septic embolization, especially to the CNS [19–21].

Haemodynamic instability secondary to valvular insufficiency, characterised by severe LV dysfunction, refractory pulmonary oedema, and/or cardiogenic shock, is the most common indication for urgent surgery. Early surgical intervention is considered where the infection spreads beyond the annulus, or BIE is complicated by perivalvular abscess or fistula, heart block, or pseudoaneurysm formation. Moreover, urgent surgery may be considered in patients with large (> 10 mm), mobile vegetations with the aim of preventing potentially catastrophic embolism [19–21]. In a randomised study, early surgical intervention, in patients with NVE and large (> 10 mm) vegetations, was associated with significant reduction in embolic events and all-cause mortality [17].

An isolated vegetation, single scallop or leaflet involvement, less extensive valve damage are factors that favour MVr as compared with replacement. No randomised studies have compared mitral valve repair with replacement in patients with NVE. However, Feringa et al. reported that the in-hospital mortality (2.3% vs. 14.4%) and long-term survival (7.8% vs. 40.5%) were significantly lower among patients who had undergone MVr as compared with replacement. In addition, the rates of reoperation, stroke, and recurrent endocarditis was also significantly lower after MVr [18].

## Conclusions

A high index of clinical suspicion is needed to promptly diagnose *H. parainfluenzae* endocarditis. It has a predilection for young women with predispositions such as bicuspid aortic valve or mitral valve prolapse. However, in this case, the patient had no identifiable risk factors. *H. parainfluenzae* endocarditis presents insidiously, and with large, destructive vegetations on the mitral valve with a propensity for systemic embolization. Cephalosporin therapy is effective and can be lifesaving.

## Data Availability

Not applicable.

## Abbreviations

AMVL: Anterior mitral valve leaflet
BIE: Bacterial infective endocarditis
CPB: Cardiopulmonary bypass
MR: Mitral regurgitation
MVr: Mitral valve repair
NVE: Native valve endocarditis
PMVL: Posterior mitral valve leaflet
PVE: Prosthetic valve endocarditis
TOE: Transoesophageal echocardiography
TTE: Transthoracic echocardiography
